# Evaluation of bee pollen by characterizing its botanical origin, total phenolic content, and microbial load for the formulation of apitherapy products

**DOI:** 10.1371/journal.pone.0327480

**Published:** 2025-09-17

**Authors:** Aysan Rezazadeh, Ahmad Reza Mehrabian, Hadi Maleki, Zahra Shakoori, Narges Zarei Golbaghi, Tayeb Sharifi, Hasan Yazdi, Mostafa Zarqami Amirsalari, Helia Hajihassani

**Affiliations:** 1 Department of Cell and Molecular Biology, Faculty of Life Sciences and Biotechnology, Shahid Beheshti University, Tehran, Iran; 2 Department of Plant Sciences and Biotechnology, Faculty of Life Sciences and Biotechnology, Shahid Beheshti University, Tehran, Iran; 3 Bee Products Research Center, Shahid Beheshti University, Tehran, Iran; 4 Department of Microbiology and Microbial Biotechnology, Faculty of Life Sciences and Biotechnology, Shahid Beheshti University, Tehran, Iran; PMAS Arid Agriculture University: PMAS-Arid Agriculture University Rawalpindi, PAKISTAN

## Abstract

Bee pollen (BP) is a beehive product known for its therapeutic properties. In this study, we aimed to evaluate 38 BP samples harvested from January 2022 to September 2022 at apiaries located in 6 provinces of Iran. The botanical origin of BP samples was determined using Scanning Electron Microscopy (SEM). The total phenolic content (TPC) of BP samples was evaluated using the Folin_Ciocalteu method. The total aerobic mesophiles were observed using Plate Count Agar (PCA). Based on the predominant grains, most samples were classified as monofloral, identifying 23 plant families. The results indicated a direct relationship between the increased abundance of plant families Asteraceae, Brassicaceae, Rosaceae, and Caryophyllaceae and the increased amount of TPC in BP samples, with the most significant positive influence of the Brassicaceae plant family. The mean value of TPC of methanol extracts of BP samples was 18.48 ± 1.97 mg of gallic acid equivalents per g (GAE/g). The mean value of the total aerobic mesophiles was 23,668.12 colony-forming units per g (CFU/g). The results were based on hygiene standards for human consumption. The current study is considered the first step toward standardizing Iranian BP.

## 1. Introduction

Apicultural products have long been used in botanical medicine and dietary practices, gaining prominence due to their beneficial health properties [1,2]. Bee pollen (BP) is one of the most essential beehive products. It is made by mixing flower pollens with bee’s salivary secretions and nectar [3], and is essential for the proper growth and development of honey bee colonies, acting as their primary protein source [4,5]. Additionally, It can meet the requirements of humans, containing a variety of nutrients: carbohydrates (13–55% of dry weight), proteins (10–40% of dry weight), lipids (1–13% of dry weight), dietary fiber and pectin (0.3–20% of dry weight), phenolic compounds (up to 2.5% of dry weight), minerals, and vitamins [6]. It is recognized as a perfect food as it contains all the essential amino acids [7] and minerals [8] necessary for the human body. In addition to its high nutritional value, BP is known to have various therapeutic applications. So far, studies have reported antioxidant [5,9–14], anti-inflammatory [10,15,16], antibacterial [11,12,17,18], and hypoglycemic [19] activity of BP, as well as its antiproliferative effects on cancer cells [20–22]. As suggested by Naseri et al., it can be used as a complementary agent for Polycystic Ovary Syndrome [23,24] and as a scaffold because it enhances bone regeneration [25]. Among the bioactive components of BP, phenolic compounds, including flavonoids and phenolic acids, play a crucial role in its antioxidant activities [26,27]. These compounds have been found to be responsible for BP’s strong radical-scavenging capacity and protective effects against electrophiles and Reactive Oxygen Species (ROS), primarily through hydrogen atom donation to neutralize free radicals. [28–30]. BPs are not equal regarding their health benefits [31], their nutritional and phenolic compounds can vary depending on their botanical and geographical origin, climatic conditions, soil characteristics, and beekeeping practices [32,33]. In addition to its bioactive components, BP contains a diverse microbial community, including both beneficial and pathogenic microorganisms, which can also vary across climatic zones [34]. Since there is a wide variety of BP products, proper quality assessment is required for commercializing them [22]. Recognizing the importance of these efforts, countries such as Lithuania, Spain, Croatia, Portugal, Morocco, Romania, Turkey, Bulgaria, Colombia, Greece, and Brazil have initiated studies to evaluate BP. However, no studies have focused on standardizing and commercializing BP In Iran. Addressing this gap is crucial for advancing the beekeeping industry and enhancing exports. In the current study, 38 BP samples were collected from 23 plant families across six provinces in Iran to investigate their botanical origin, Total Phenolic Content (TPC), and microbial composition. The employed methodologies included palynological identification and Scanning Electron Microscopy (SEM) for botanical analysis, spectrophotometric measurement for TPC, and microbial assessments. Statistical analyses were conducted to explore correlations between botanical origin, TPC, and microbial profiles. These findings aim to enhance understanding of the quality and safety of Iranian BP, contributing to its standardization and commercialization.

## 2. Materials and methods

### 2.1. Bee pollen (BP) sample collection and palynological identification

38 BP samples were collected by professional beekeepers, and some were purchased from bee keepers’ shops from January 2022 to September 2022 at apiaries located in different provinces of Iran. This country, with diverse climatic and geographical zones, is the habitat of more than 8,000 plant species. At least 2,300 of them possess aromatic and medicinal properties [26]. This significant botanical diversity across Iran turns it into our study area.

**Fig 1:**
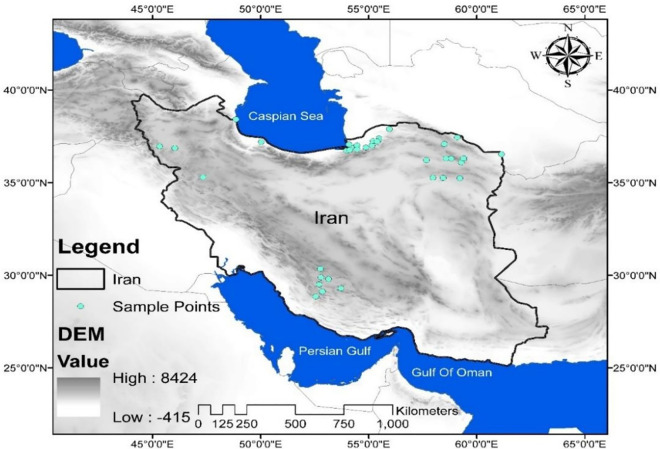
Geographical locations of 38 BP samples collected from 6 provinces of Iran.

Fig 1 provides detailed information about the geographical location of the BP samples.

BP samples were transferred directly to the laboratory and stored in the refrigerator at 4°C until analysis. All the samples were handled aseptically to prevent secondary contamination. In the laboratory, 2 g of BP grains were separated based on their color, according to the methodology suggested by Almeida et al. (A 2 g sample, approximately equivalent to 300 BP grains, was considered representative for pollen analysis) [27]. The botanical origin of BP was assessed by comparing its physical characteristics—such as morphology, size of polar and equatorial axis, viscin threads, diameter of pore, arms, and exine ornamentations—using Scanning Electron Microscopy (SEM), atlases, international palynology websites, and pollen data compiled by the research group at Shahid Beheshti University [28–30].

### 2.2. Scanning electron microscopy (SEM)

BP grains were extracted from mature anthers and mounted on aluminum stubs, then coated with gold in an Emitech EMK 550 sputter. Image tool ver. 30 was used to assess quantitative BP characteristics; They were coded as multistate characteristics and used for cluster and PCOA analyses. PAST software was used for numerical analyses [31,32]. Based on Louveaux et al. the following terms were used for classifying the percentage of BP grains: “predominant pollen grains” (>45% of total); “secondary pollen grains” (16–45% of total); “important minor pollen” (3–15% of total), and “minor pollen” (<3% of total) [33]. Observations were conducted using a scanning electron microscope (SEM) Hitachi SU3500 electron microscope at Shahid Beheshti University.

### 2.3. Total phenolic content (TPC)

The TPC of BP samples was analyzed using the Folin_Ciocalteu colorimetric method, described by Singleton et al. [35]. Briefly, 0.2 g of each BP sample was diluted in 5 mL methanol solution (70% v/v), followed by incubation in a water bath for 10 minutes at the temperature of 70 ⁰C. After cooling, it was poured into centrifuge tubes for centrifugation at 3,500 rpm for 10 minutes using a Gerber Centrifuge (FUNKE GERBER, Germany). The supernatant was carefully removed. Finally, the extract was mixed with 5 mL of Folin–Ciocalteu reagent (10% v/v), and after 3–8 minutes, 4 mL of sodium carbonate solution (7.5% w/v) was added. The absorbance was measured at 765 nm after 1 hour of incubation using a UV-2100 Spectrophotometer (UNICO, China). The absorbance was determined against the blank (containing only distilled water and reagent). Gallic acid standard solutions (10–50 mg/ml) were used for constructing the calibration curve $\left(y=0.132x+0.0113;\;R^{2}=0.9985\right)$. The results are expressed as mg of gallic acid equivalent per g (GAE/g) of BP samples [36].

### 2.4. Microbial analysis

The aerobic mesophiles were determined using Plate Count Agar (PCA). 5 g of each BP sample was added to 45 ml of standard saline solution and vortexed. Serial dilutions were prepared to 10^−5^. Using the pour plate technique, 1 ml of each aliquot concentration was inoculated into PCA. The plates were incubated at 37 °C for 24 hours, after which colonies were counted. The results are reported as colony-forming units per g (CFU/g) of the BP sample [37].

### 2.5. Statistical analysis

All the analyses were performed in duplicate unless stated otherwise, and the results are expressed as the mean value ± standard deviation (SD), calculated using SPSS v.26 statistical package software for Windows. A 95% confidence level linear regression analysis was performed to assess the relationship between TPC and each plant family. To evaluate the variation in the abundance of effective plant families, a one-way analysis of variance (ANOVA) was employed, also at a 95% confidence level, using Minitab 19. Furthermore, the TPC of BP collected from various geographical locations was visually represented using ArcGIS 10.8 software, mapping the distribution across the study area.

## 3. Results

### 3.1. Botanical identification of bee pollen (BP) using scanning electron microscopy

**Fig 2:**
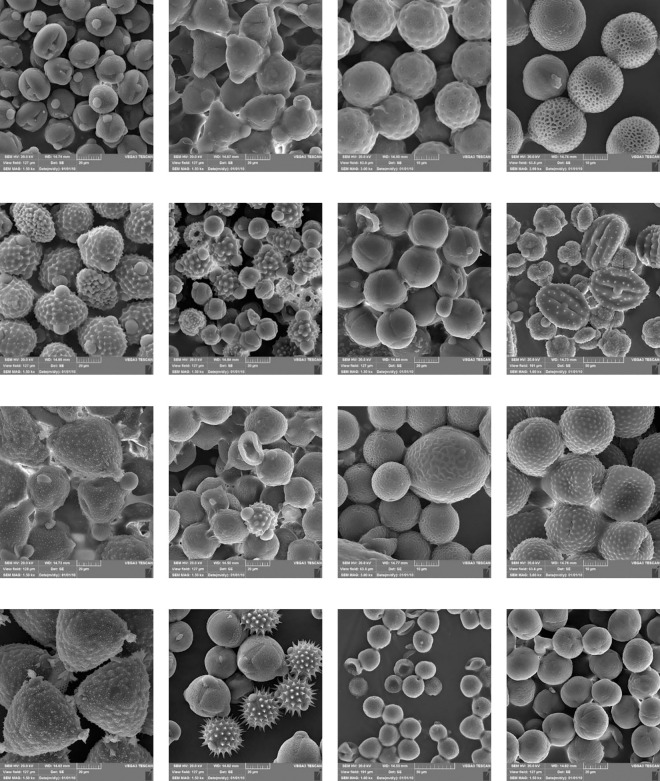
Scanning Electron Microscopy (SEM) micrographs of BP samples.

A full spectrum palynological analysis of the BP samples is provided in Fig 2.

23 plant families were identified. BPs were analyzed according to their predominant grains. All the samples but BP8, BP9, BP11, BP18, BP20, BP26, BP27, BP36, and BP38 were classified as monofloral. None of the botanical families were consistently recognized across all the analyzed BP samples; this variation is influenced by the vegetation surrounding the apiary, particularly in our study area. Tables 1 and 2 verify detailed information.

**Table 1:** Palynological spectrum (frequency (%)) of plant families (botanical origin) obtained in BP samples.

Family	List of BP samples and their frequency (%)																		
	BP1	BP2	BP3	BP4	BP5	BP 6	BP7	BP8	BP9	BP10	BP11	BP12	BP13	BP14	BP15	BP16	BP17	BP18	BP19
Amaranthaceae	5 **IP**									11 **IP**	2 **MP**								
Apiaceae								4 **IP**											
Asteraceae	20 **SP**		28 **SP**	70 **PP**		13 **IP**	32 **SP**	24 **SP**		53 **PP**	44 **SP**	35 **SP**	14 **IP**		70 **PP**	17 **SP**	57 **PP**	19 **SP**	62 **PP**
Boraginaceae		5 IP															28 **SP**		
Brassicaceae	55 **PP**	71 **PP**	51 **PP**	17 **SP**		75 **PP**					23 **SP**						1 **MP**	26 **SP**	
Caprifoliaceae			3 IP					22 **SP**											
Caryophyllaceae								4 **IP**	20 **SP**		8 **IP**					17 **SP**	1 **MP**		
Cleomaceae																		19 **SP**	
Dipsacaceae																1 **MP**			
Fabaceae			16 **SP**	12 **IP**			52 **PP**	24 **SP**	40 **SP**			51 **PP**	85 **PP**			14 **IP**	13 **IP**		26 **SP**
Gymnosperm																			12 **IP**
Lamiaceae			1 **MP**	1 **MP**				22 **SP**	36 **SP**										
Malvaceae					70 **PP**									70 **PP**					
Papaveraceae																			
Plantaginaceae	20 **SP**	14 **IP**					10 **IP**			36 **SP**	21 **SP**		1 **MP**					36 **SP**	
Poaceae																			
Rhamnaceae		10 **IP**																	
Rosaceae						11 **IP**			4 **IP**										
Rutaceae							1 **MP**					14 **IP**							
Sapindaceae																51 **PP**			
Scrophulariaceae			1 **MP**				5 **IP**												
Solanaceae											2 **MP**								
Violaceae						1 **MP**													

PP = predominant pollen grains (>45% of total); SP = secondary pollen grains (16–45% of total); IP = important minor pollen (3–15% of total); and MP = minor pollen grains (<3% of total).

**Table 2:** Palynological spectrum (frequency (%)) of plant families (botanical origin) obtained in BP samples.

Family	List of BP samples and their frequency (%)																		
	BP20	BP21	BP22	BP23	BP24	BP25	BP26	BP27	BP28	BP29	BP30	BP31	BP32	BP33	BP34	BP35*	BP36	BP37	BP38
Amaranthaceae																			
Apiaceae		21 **SP**																	
Asteraceae	30 **SP**	53 PP		2 **MP**	70 **PP**	18 **SP**		41 **SP**	70 **PP**	61 **PP**					70 **PP**		41 **SP**		14 **IP**
Boraginaceae																			12 **IP**
Brassicaceae	16 **SP**	3 **IP**				47 **PP**	40 **SP**	36 **SP**		39 **SP**		61 **PP**					33 **SP**	70 **PP**	20 **SP**
Caprifoliaceae																			
Caryophyllaceae	18 **SP**	11 **IP**												70 **PP**					
Cleomaceae																			
Dipsacaceae							30 **SP**												
Fabaceae	36 **SP**					3 **IP**	30 **SP**												18 **SP**
Gymnosperm																			
Lamiaceae																			
Malvaceae																			
Papaveraceae		6 **IP**																	
Plantaginaceae																			
Poaceae											35 **SP**	39 **SP**	70 **PP**						
Rhamnaceae																			11 **IP**
Rosaceae		6 **IP**	70 **PP**	96 **PP**		31 **SP**		23 **SP**			65 **PP**						26 **SP**		25 **SP**
Rutaceae				2 **MP**		1 **MP**													
Sapindaceae																			
Scrophulariaceae																			
Solanaceae																			
Violaceae																			

PP = predominant pollen grains (>45% of total); SP = secondary pollen grains (16–45% of total); IP = important minor pollen (3–15% of total); and MP = minor pollen grains (<3% of total). * = not identified.

### 3.2. Correlation between the identified plant families and the total phenolic content (TPC)

**Fig 3:**
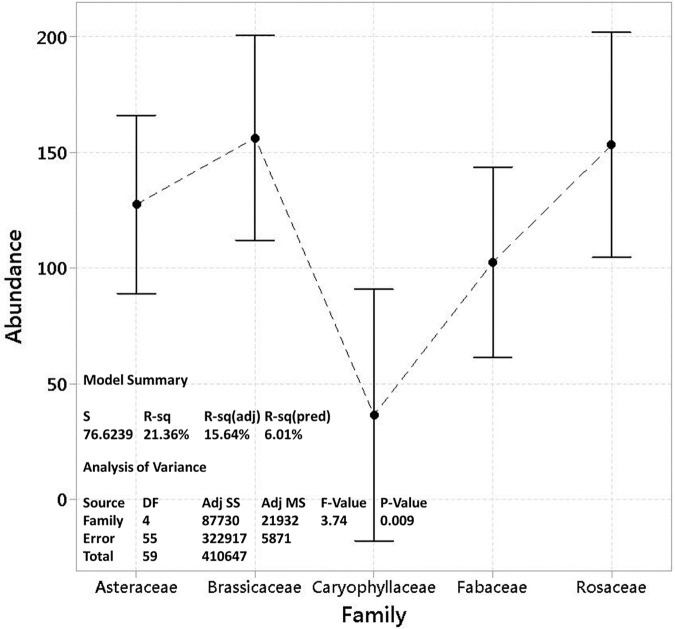
ANOVA results showing significant differences in TPC of bee pollen (BP) across five plant families.

The analysis of variance (ANOVA) revealed statistically significant differences in the frequency of the five plant families affecting the TPC in BP. The ANOVA results (F = 3.74, df = 59, p < 0.009) demonstrated that the variation in TPC is strongly associated with the selected plant families. The low p-value (p < 0.009) indicates that the differences observed among the plant families are unlikely due to chance, confirming the critical role these families play in shaping the phenolic profile of BP. Given that honey bees selectively forage on specific plant species based on nectar and pollen composition, our findings suggest that floral origin plays a key role in determining BP’s chemical properties. While our study does not directly analyze bee foraging behavior, the significant variation in TPC across different plant families is consistent with the established influence of floral origin on BP composition (Fig 3).

**Fig 4:**
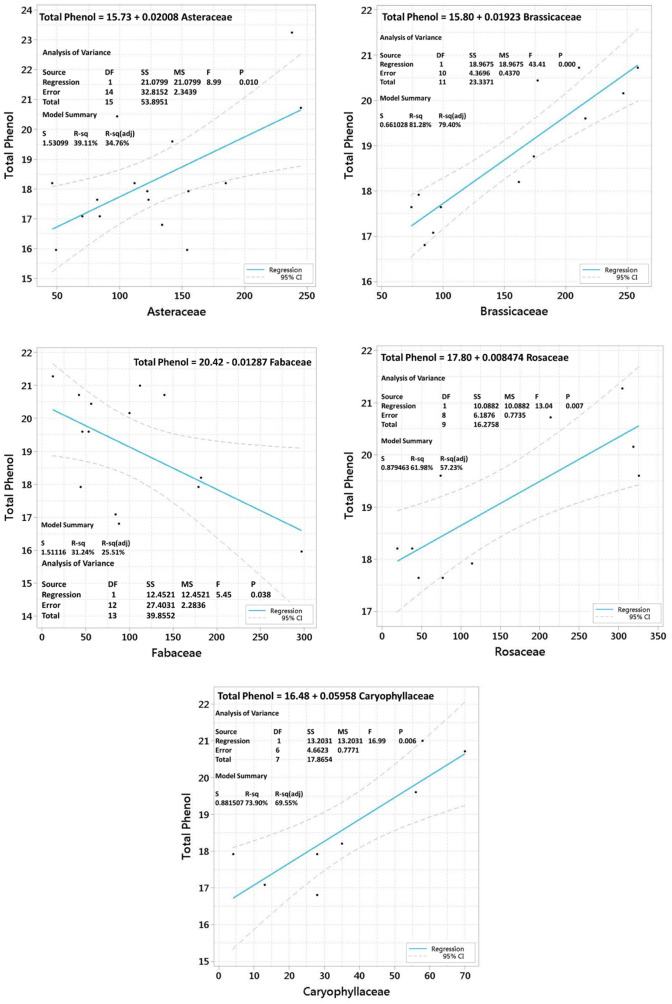
Plant families analyzed for their correlation with TPC in BP.

Among the identified plant families, Asteraceae (p 0.010, R^2^ > 39.11%), Brassicaceae (p 0.000, R^2^ > 81.28%), Fabaceae (p 0.038, R^2^ > 31.24%), Rosaceae (p 0.007, R^2^ > 61.98%), and Caryophyllaceae (p 0.006, R^2^ > 73.90%), were respectively the most influential plant families in the TPC of the BP samples. The results indicated a direct relationship between the increased abundance of plant families Asteraceae, Brassicaceae, Rosaceae, and Caryophyllaceae and the increased TPC in BP samples (Fig 4).

**Fig 5:**
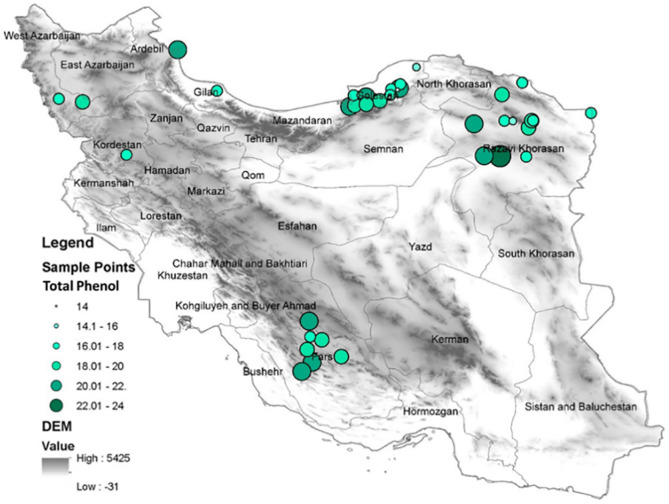
The range of TPC of BP in the sampled areas.

The analysis of TPC in the studied samples indicates that geographical location significantly influences phenolic levels. Fig 5 illustrates the variation in the TPC of BP across the sampled geographical locations.

#### 3.2.1. Microbial analysis.

Table 3 represents all the samples but BP21, BP29, and BP33 possessed acceptable microbiological quality. The average count of the total aerobic mesophiles was 23,668.12 CFU/g. The high microbiological quality in the mentioned samples might be according to their botanical origin, environmental conditions, processing, and handling as the secondary contamination [37,38].

**Table 3:** Microbial analysis of the BP, (*represents estimated APC).

Sample	Total mesophilic count (CFU/g)
BP1*	1.5 × 10^1^
BP2	2.2 × 10^2^
BP3	8.0 × 10^3^
BP4	4.2 × 10^3^
BP5*	0.5 × 10^1^
BP6	6.8 × 10^2^
BP7	3.2 × 10^3^
BP8*	<1.0 × 10^1^
BP9*	7.0 × 10^2^
BP10	4.4 × 10^2^
BP11*	2.5 × 10^1^
BP12	3.6 × 10^2^
BP13	5.0 × 10^2^
BP14	1.4 × 10^2^
BP15*	2.0 × 10^3^
BP16*	5.0 × 10^3^
BP17*	3.0 × 10^3^
BP18*	4.0 × 10^4^
BP19*	1.0 × 10^4^
BP20*	4.5 × 10^3^
BP21	1.4 × 10^5^
BP22*	1.5 × 10^3^
BP23	4.0 × 10^4^
BP24*	3.0 × 10^3^
BP25*	2.0 × 10^3^
BP26*	2.0 × 10^3^
BP27	2.0 × 10^4^
BP28	9.7 × 10^4^
BP29	2.3 × 10^5^
BP30	3.8 × 10^4^
BP31	3.0 × 10^3^
BP32	6.5 × 10^4^
BP33	1.2 × 10^5^
BP34	1.8 × 10^3^
BP35	2.5 × 10^3^
BP36	4.5 × 10^4^
BP37	2.6 × 10^3^
BP38	3.0 × 10^3^

## 4. Discussion

### 4.1. Palynological identification

The full spectrum palynological analysis confirms that Iran possesses a wide range of botanical diversity. The most common predominant plant families in BP samples were *Asteraceae* (26 samples, 22.03% of the total), *Brassicaceae* (18 samples, 15.25% of the total), *Fabaceae* (14 samples, 11.86% of the total), *Rosaceae* (10 samples, 8.47% of the total), and *Caryophyllaceae* (8 samples, 6.78% of the total). Our previous study examined the amount of antioxidant activity in honey. We recognized an increase in the abundance of plant families Rosaceae, Asteraceae, Amaranthaceae, and Fabaceae increases the amount of antioxidant activity of the studied honey samples, so we can conclude that common botanical families represent the effective flora present in Iran [34,39]. BP samples predominantly from the Brassicaceae plant family possessed higher phenolic compounds than other plant families (p 0.000, R2 > 81.28%). Members of this diverse plant family are known for their medical properties in treating cardiovascular diseases, diabetes, obesity, and various cancers [40–42]. They are rich in phenolic compounds, carotenoids, and glucosinolates that are associated with their nutritional properties [40,42].

### 4.2. Total phenolic content (TPC) analysis and its correlation with identified plant families

Phenolic compounds are secondary metabolites synthesized by specialized plant cells [43]. Since human body cells lack this ability, they must be obtained through the diet to fulfill this need [44]. Interestingly, phenolic compounds are considered the diet’s most abundant antioxidants, which can neutralize the reactive oxygen and nitrogen species by accepting electrons, thereby disrupting chain oxidation reactions [45,46]. They possess several medical implications [47]; they act as anti-cancer, anti-diabetes, anti-aging, and anti-inflammatory agents, preventing chronic diseases [48,49]. However, detailed information from databases containing BP profiles must be used to determine whether BP possesses inhibitory activity against cancer lines [50–52]. The TPC ranged from 14.00 to 23.24 (mean value ± SD = 18.48 ± 1.97 GAE/g). The TPC values of the current study were above the value summarized by Campos et al. as an international directive for quality control of BP (0.23 ± 0.02% GAE/g) [53]. Similar results were obtained in the study by Čeksteryte et al. in Lithuania (23.3 ± 0.1 GAE/g) [54], while lower values were reported by Feás et al. in Spain (16.4 ± 2.0 GAE/g) [55], and by Bilić Rajs et al. in Croatia, where the results ranged from 4.0 to 15.8 GAE/g [56]. However, higher values were obtained by Pascoal et al. in the northeast of Portugal, (26.71 ± 6.19 GAE/g) in Mogadouro and (22.75 ± 5.04 GAE/g) in Vimioso [44], by El Ghouizi et al. in Morocco (21.87 ± 1.80 GAE/g) [5], by Spulber et al. in Romania with highest value of 25.33 GAE/g [57], and by Mayda et al. in Turkey, where the results ranged from 8.26 to 43.42 GAE/g [58]. Many studies have recognized a positive correlation between phenolic compounds and antioxidant activity, as well as the botanical and geographical origin of BPs [59,60]. Feás et al. recognized a correlation coefficient (R = 0.6) between the TPC of BP and DPPH radical scavenging activity [55]. Similar results were contributed by El Ghouizi et al [5].

### 4.3. Influence of harvesting time

As mentioned before, the TPC of BP varies according to climatic conditions [61]. Negrão et al. recognized that the harvesting season influences BP production and its nutritional composition. They concluded that winter is the most favorable season [62]. Our study’s harvesting period was from January 2022 to September 2022. It partially happened during the period, but harvesting in the winter is highly recommended for yielding better quality. It remains difficult to predict the exact potential therapeutic application of BP samples without a detailed analysis.

### 4.4. Microbial quality

The average count of the total aerobic mesophiles was 23,668.12 CFU/g, which is lower than the value of 100,000 CFU/g described by Campos et al. [63], so all the samples but BP21, BP29, and BP33, met the hygienic standards. The total aerobic mesophiles obtained in this study were lower than those harvested by Beev et al. in Bulgaria, which ranged from 400 to 67,000 CFU/g [64], and by Vargas-Abella et al. in Colombia, which ranged from 9,000–12,000 CFU/g [65]. Additionally, lower the study reported by Liolios et al. in Greece [66]. However, the results were significantly higher than those harvested by Anjos et al. in Portugal, which ranged from 35.7 to 1,390 CFU/g [67], by Peixoto et al. in Portugal which ranged from 10 to 3,000 CFU/g [68], and by Santa Bárbara et al. in Brazil which ranged from 85 to 443 CFU/g [69]. On the other hand, our results were comparable to the study by Sevin et al. in Turkey [70]. The total aerobic mesophiles were counted because microbial safety plays a critical role in the acceptability of foodstuffs, but destructive methods have failed to achieve acceptable outcomes, as they risk generating toxic residues [71]. Recently, Chen et al. developed a combined treatment technology for pathogen inactivation in BP, integrating drying, ethanol exposure, and atmospheric cold plasma. This novel method enhances microbial inactivation and preserves the BP nutrients [72]. This data cannot confirm the suitability of BP for human consumption, because we only count the total aerobic mesophiles. According to the hygienic standards, additional tests must be performed to determine whether these samples exhibit microbial contamination, and in the current study despite covering major beekeeping hubs, the whole country was not covered, So the exact potential of Iran is still underestimated. Novel techniques like DNA Metabarcoding are highly recommended to monitor which genes are responsible for phenolic compounds, by using this method the resistance genes toward some pathogens might be identified.

## 5. Conclusion

BP is significantly influenced by its botanical and geographical origin, so precise evaluation is needed for each country to recognize high-quality BP. In the current study, we assessed palynological and microbial analysis as well as the TPC of BP for the first time in Iran. Most of the samples met the microbial quality criteria. We identified the direct correlation between the TPC and the botanical origin of the BP. The Brassicaceae family had the most significant positive influence on the TPC compared to the other plant families. We aimed to enrich the knowledge of BP among beekeepers, consumers, and policymakers. As mentioned above this research is the first reference in standardizing the BP produced in Iran. Ultimately, these assessments will serve as a detailed screening for selecting raw materials for the formulation of apitherapy products.
